# Increasing phosphorus supply is not the mechanism by which arbuscular mycorrhiza increase attractiveness of bean (*Vicia faba*) to aphids

**DOI:** 10.1093/jxb/eru283

**Published:** 2014-07-08

**Authors:** Zdenka Babikova, Lucy Gilbert, Kate C. Randall, Toby J. A. Bruce, John A. Pickett, David Johnson

**Affiliations:** ^1^Institute of Biological and Environmental Sciences, University of Aberdeen, Cruickshank building, St Machar Drive, Aberdeen AB24 3UU, UK; ^2^James Hutton Institute, Craigiebuckler, Aberdeen AB15 8QH, UK; ^3^Rothamsted Research, Harpenden, Hertfordshire AL5 2JQ, UK

**Keywords:** Arbuscular mycorrhizal fungi, broad bean (*Vicia faba*), insect host location, pea aphid (*Acyrthosiphon pisum*), phosphorus, plant volatiles.

## Abstract

Arbuscular mycorrhizal (AM) fungi, important plant mutualists, provide plants with nutrients such as phosphorus (P) in return for carbon. AM fungi also enhance the attractiveness of plants to aphids via effects on emissions of plant volatiles used in aphid host location. We tested whether increased P uptake by plants is the mechanism through which AM fungi alter the volatile profile of plants and aphid behavioural responses by manipulating the availability of P and AM fungi to broad beans (*Vicia faba* L.) in a multi-factorial design. If AM fungi affect plant volatiles only via increased P acquisition, we predicted that the emission of volatiles and the attractiveness of mycorrhizal beans to aphids would be similar to those of non-mycorrhizal beans supplied with additional P. AM fungi and P addition increased leaf P concentrations by 40 and 24%, respectively. The production of naphthalene was less in mycorrhizal plants, regardless of P addition. By contrast, production of (*S*)-linalool, (*E*)-caryophyllene and (*R*)-germacrene D was less in plants colonized by AM fungi but only in the absence of P additions. The attractiveness of plants to pea aphids (*Acyrthosiphon pisum* Harris) was positively affected by AM fungi and correlated with the extent of root colonization; however, attractiveness was neither affected by P treatment nor correlated with leaf P concentration. These findings suggest that increased P uptake is not the main mechanism by which mycorrhiza increase the attractiveness of plants to aphids. Instead, the mechanism is likely to operate via AM fungi-induced plant systemic signalling.

## Summary

Increasing phosphorus supply is not the mechanism by which arbuscular mycorrhiza increase attractiveness of bean, *Vicia faba*, to aphids.

## Introduction

Arbuscular mycorrhizal (AM) fungi colonize the roots of plants in ~80% of land plant families (Johnson *et al.*, 2005; Gosling *et al.*, 2006) and are ubiquitous within natural and agricultural ecosystems (Smith and Read, 2008). The symbiosis provides benefits for plants in increased acquisition of mainly phosphorus (P) and water (Smith and Read, 2008) and other nutrients (Lambers *et al.*, 2008), and resistance against biotic (Van Wees *et al.*, 2008) and abiotic (Maya and Matsubara, 2013) stresses. In return, AM fungi gain carbohydrates from the plant (Smith and Read, 2008), which are used to form extensive extraradical mycelial networks. These networks enable a much larger soil volume to be explored for nutrients, particularly P (Smith and Read, 2008). This process is important because, in most ecosystems, P is an essential growth-limiting macronutrient for plants (Wassen *et al.*, 2005). Therefore, the productivity and leaf P concentration of many plants in P-deficient soils is dependent on their mycorrhizal status (e.g. Ortas, 2012).

It is well established that AM fungi greatly influence the outcome of plant–herbivore interactions (Gange *et al.*, 1999; Hartley and Gange, 2009; Koricheva *et al.*, 2009). One of the most abundant and agriculturally important groups of invertebrate herbivores on above-ground parts of plants is aphids (Dixon, 1977). Aphids have a sophisticated mechanism to feed on carbohydrates and amino acids directly from the phloem, thereby avoiding defence chemicals from the leaf tissue. They alternate between sexual and asexual generations, which allows them to proliferate rapidly (Guerrieri and Digilio, 2008), and can suppress defence compounds released by plants (Schwartzberg *et al.*, 2011). Plant sap-sucking insects are generally positively affected by the presence of AM fungi on roots, because the fungi enhance the quality of host plants, which is principally a result of increased nitrogen (N) and P concentrations (Gange *et al.*, 1999; Koricheva *et al.*, 2009; Hartley and Gange, 2009).

In addition to their effects on plant nutrition, AM fungi also influence plant defence metabolism (Pozo and Azcón-Aguilar, 2007), including biosynthesis of plant volatile organic compounds (VOCs). Consequently, mycorrhizal plants emit different VOCs compared with non-mycorrhizal plants (Nemec and Lund, 1990; Fontana *et al.*, 2009; Babikova *et al.*, 2013*a*). Specific VOCs act as cues for host-locating herbivores including aphids (Bruce *et al.*, 2005) and their enemies, parasitoid wasps, which are attracted to plants via herbivore-induced VOCs (De Moraes *et al.*, 1998). Therefore, AM fungi–plant interactions may influence host location responses of insects, mediated via VOCs (Guerrieri *et al.*, 2004; Cosme *et al.*, 2011; Schausberger *et al.*, 2012; Babikova *et al.*, 2013*a*). However, the underlying mechanism by which AM fungi induce changes in volatile emission and hence alter the attractiveness of plants to aphids is not yet understood.

Because VOCs can be affected by abiotic factors (Gouinguené and Turlings, 2002) including soil nutrient availability (Van Der Putten *et al.*, 2001; Ormeño *et al.*, 2008; Delgado-Rodríguez *et al.*, 2011; Veromann *et al.*, 2013) and mycorrhizal plants often contain more P in their tissues than non-mycorrhizal plants (Vannette and Hunter, 2009; Ryan *et al.*, 2012; Walder *et al.*, 2012), it is possible that the mechanism of AM fungi-induced changes in VOC emission operates through increased P uptake. This mechanism could have implications for management of agroecosystems, where the practice of fertilizer use is common. However, P availability also affects the dependence of plants on AM fungi: the symbiosis is more common in P-limited soils (Smith and Read, 2008), while plants are often less likely to form mycorrhizal associations in soils with high concentrations of plant-available P (Smith and Read, 2008; Ortas, 2012; Gosling *et al.*, 2013). We therefore need to better understand the role of P availability on volatile emission and whether it affects the host-locating abilities of herbivorous insects such as aphids and their natural enemies.

In this study, we investigated the mechanisms for the increased attractiveness of mycorrhizal plants to aphids by experimentally manipulating the availability of both P and AM fungi to plants. We tested the hypothesis that AM fungi-induced changes in VOCs emission operate mainly through increased P uptake. To test this hypothesis, we conducted an experiment with broad beans (*Vicia faba* L.) using multi-factorial treatments of AM fungi (–/+) and P addition (–/+).

If AM fungi affect aphid development and the attractiveness of plants to aphids mainly via changes in plant P acquisition, we predicted that the attractiveness of plants to aphids and emission of VOCs would be the same when beans were colonized by AM fungi compared with non-mycorrhizal beans supplied with elevated amounts of P. If the hypothesis was not supported, and VOC induction operated through other aspects of association with AM fungi, we predicted that AM fungi would enhance the attractiveness of plants to aphids both with and without P addition, whereas non-mycorrhizal plants would be unattractive to aphids regardless of P treatment. We quantified production of plant VOCs in response to treatments and tested the attractiveness of plants to aphids in behavioural assays. A subset of the plants was infested with pea aphids (*Acyrthosiphon pisum* Harris) enabling us to test for effects of AM fungi and P treatments on aphid biomass. We also measured the extent of mycorrhizal fungal colonization in response to aphids and P treatment.

## Materials and methods

### Plants, soil, fungi and aphids

Seeds of broad bean (*V. faba*) cultivar ‘The Sutton Dwarf’ (Moles seeds, Colchester, UK) pre-germinated for 5 d were grown in 1-litre pots containing a soil mixture. The soil mixture consisted of 1/3 sand, 1/3 horticultural grit, and 1/3 loamy top soil [all nutrients solely from the base materials: 9% clay, 17% silt, 74% sand, pH 7.8, organic matter 24.2%, total nitrogen (Dumas) 0.74%, available phosphorus 64mg l^–1^, available potassium 1324mg l^–1^, available magnesium 222mg l^–1^], all from LBS (Colne, UK). The composition of the potting mixture was nutrient poor.

The AM fungal inoculum (BioOrganics LLC, Santa Maria, CA, USA) consisted of spores of eight species (*Glomus aggregatum*, *Glomus clarum*, *Glomus deserticola*, *Glomus monosporus*, *Glomus mosseae*, *Rhizophagus irregularis*, *Gigaspora margarita and Paraglomus brasilianum*) in clay powder carrier (approximately 50 spores ml^–1^). The inoculum was bulked using *Plantago lanceolata* L. to provide a final inoculum comprising sand, spores, and roots. A subsample of this inoculum was autoclaved, which was added to non-AM fungal treatments ensuring all plants were subjected to same soil potting composition. Both autoclaved and live inoculum was placed as a layer in the lower part of the pots.

A clone of pea aphid (*A. pisum*) from Rothamsted Research (Harpenden, UK) was maintained in the laboratory at 20 °C and 16h light on broad beans, the same cultivar as the experimental plants.

### Experimental design

The experiment consisted of three treatments each with two levels that were applied in all possible eight combinations (*n*=6) (see Table 1). The availability of P was manipulated by watering soil in the pots with Hoagland’s solution, which contained either no added P or twice the concentration of KH_2_PO_4_. For the +P treatment, the solution contained 1.65mM Ca(NO_3_)_2_, 0.51mM KNO_3_, 0.49mM MgSO_4_.7H_2_O, and 0.54mM KH_2_PO_4_. The amount of P added was equivalent to 90kg ha^–1^ of P_2_O_5_, which is typical of fertilizer additions in agricultural systems (Hashemabadi, 2013). All stock solutions were prepared using chemicals from Fisher Scientific UK Ltd. Plants were watered with 300ml of solutions given in three applications per week for the entire duration of the experiment. When necessary, plants were watered additionally with distilled water. The pots were randomized in a glasshouse (25±7 °C).

**Table 1.** Effects that we predicted treatments would have on plant nutrition and growth parameters, VOCs, attractiveness of plants to aphids, percentage of root length colonized by AM fungi, and final aphid biomass in relation to treatment group (aphids –/+, AM fungi –/+, P –/+), based on the hypothesis that AM fungi enhance attractiveness of plants to aphids due to increased P uptakeThe predicted effect is indicated by an arrow (↑, positive effect; →, no effect; ↓, negative effect; two arrows indicate a stronger effect). The arrows represent the comparison with the first applicable treatment in the row, so the first arrow in each row is always → (no effect). The same letters indicate that composition of plant volatiles is predicted to be similar. NA, not applicable.

	Treatment								
	AM fungi	–	+	–	+	–	+	–	+
	P	–	–	+	+	–	–	+	+
	Aphids	–	–	–	–	+	+	+	+
Leaf P		→	↑	↑	↑	↓	→	→	→
Leaf N		→	↑	→	↑	↓	→	↓	→
Plant biomass		→	↑	↑	↑	↓	→	→	→
VOCs		A	B	B	B	NA	NA	NA	NA
Attractiveness		→	↑	↑	↑	NA	NA	NA	NA
Colonization		NA	→	NA	↓	NA	↓	NA	↓↓
Aphid biomass		NA	NA	NA	NA	→	↑	↑	↑

Six weeks after planting, four adult aphids of the same weight were added to half of the plants from each treatment group and all experimental plants were enclosed in nets to prevent cross-contamination. The development of aphids was recorded by weighing the total mass of aphids, which were brushed from plants at harvest and dried in an oven at 60 °C for 48h.

### Measurements of plant biomass, leaf N and P concentrations, and AM fungal colonization

When plants were harvested at week 9, plant above-ground dry mass was weighed after drying at 80 °C for 48h. Plant root mass was weighed fresh. Dried and homogenized subsamples of leaves were digested with sulfuric acid and hydrogen peroxide and analysed by flow injection analysis (FIA star 5000, Foss, Denmark) to measure total P and N concentrations. We also quantified acetic acid-extractable (i.e. plant available) PO_4_ (Allen, 1989).

To measure the extent of mycorrhizal colonization, trypan blue-stained root fragments were assessed microscopically, using the magnified intersection method (McGonigle *et al.*, 1990), by scoring >100 intersects from at least three slides per sample.

### Plant headspace samples

Plants that had not been treated with aphids were used for collection of headspace samples prior to harvest using an air entrainment kit (BJ Pye, Kings Walden, UK) as described previously (Bruce *et al.*, 2008). VOCs were quantified using gas chromatography (GC) on non-polar (HP-1, 50 m×0.32mm inner diameter×0.5mm film thickness; J & W Scientific) capillary column using a HP6890 GC (Agilent Technologies, UK) fitted with a cool-on-column injector, a deactivated retention gap (1 m×0.53mm inner diameter) and a flame ionization detector. The GC oven temperature was maintained at 30 °C for 1min after sample injection and then raised by 5 °C min^–1^ to 150 °C, and then by 10 °C min^–1^ to 250 °C. The carrier gas was hydrogen. Samples (2 μl) were injected using an HP 7683 series injector. This analysis was restricted to 16 VOCs (Supplementary Table S1 at *JXB* online) identified and determined to be electrophysiologically active to pea aphid by GC-coupled electroantennography (EAG) in previous studies that used broad bean plants grown under the same conditions to those in the present work (Babikova *et al.*, 2013*b*) in which the enantiomers of the natural VOCs were also determined. Our analysis thus quantified only those VOCs that are considered biologically relevant to pea aphids. VOCs produced per plant were quantified as a percentage of peak areas compared with a series of alkanes used as external standards (Skelton *et al.*, 2010). For statistical analysis, we used the amounts of each individual EAG-active VOC calculated per unit of plant biomass (see Supplementary Table S1 available at *JXB* online).

### Attractiveness of plant headspace samples to pea aphids

We assessed pea aphid response to plant headspace samples using bioassays in a four-way olfactometer (Pettersson, 1970; Webster *et al.*, 2010) using all headspace samples collected from aphid-free plants. The experimental arena of the olfactometer consisted of a central area connected to four glass arms. Pieces of filter paper treated with reagent blanks were attached to three of the arms, while a paper treated with a solution (10 μl) of plant headspace sample (Bruce *et al.*, 2008) was attached to the remaining arm. Only morphs of aphids at the migratory (winged) stage were used in the bioassays, and these were starved in Petri dishes in the area used for the bioassays for 2–4h prior to the bioassay. The bioassay aphid was placed inside the central area and air was pulled through the apparatus by a suction pump connected at the centre with a gentle airflow (200ml min^–1^). Aphid movement in the arena was recorded on OLFA software (Udine, Italy) during the bioassay. Each bioassay was conducted for 16min, and every 2min the olfactometer was turned 45° in one direction to avoid any bias caused by uneven light. Each headspace sample (*n*=6 per treatment) was tested in five separate bioassays each using a new aphid and fresh preparations of samples and control on the filter paper.

### Data analyses

The predicted effects of treatment (according to our hypothesis that AM fungi affect aphid development and the attractiveness of plants to aphids mainly via changes in plant P acquisition) on plant nutrition and growth, production of VOCs, attractiveness of plants to aphids, percentage root length colonized, and aphid biomass are shown in Table 1.

To test for the effects of treatments on total leaf N and P concentrations, leaf N:P ratio, above-ground plant biomass, and concentration of extractable soil PO_4_, we used general linear models (GLMs) with main effects (AM fungi, P, and aphids) and all two-way and three-way interactions as fixed factors.

Using GLMs, we tested for effects of AM fungi, P treatment, and their interactions on emissions of plant VOCs using, first, the amounts of each EAG-active VOCs [g^–1^ of dry weight (DW)] as separate response variables and, secondly, the sum of all EAG-active VOCs. The entrainment day was included in the model as a random factor because the collection of VOCs was conducted over several days.

Attractiveness of headspace samples to aphids was calculated as the time spent by aphids in the olfactometer area containing solvent blanks (mean of three control areas) subtracted from the times spent in the olfactometer area containing headspace samples. Using a GLM, we tested for effects of AM fungi and P treatment and their interaction on attractiveness of plants to aphids. In addition, to test whether each individual treatment produced plants that were significantly attractive to aphids or not, the mean times spent by aphids in the treated areas from each headspace samples were compared with mean times spent in control areas (means of three control areas) for each treatment separately using paired *t*-tests (Bruce *et al.*, 2008).

To investigate how the attractiveness of plants to aphids is regulated by AM fungi and other plant characteristics, we tested for relationships between attractiveness as a response variable and the following explanatory variables: percentage of root length colonized, plant shoot and root mass, total leaf P concentration, total leaf N concentration, N:P ratio, and concentration of soil extractable PO_4_. In addition, because plant, soil, and fungal characteristics might influence the attractiveness of plants to aphids indirectly, we used a series of linear regressions to test for associations between response variables: root length colonized and soil PO_4_; total leaf P concentration and total leaf N concentration; and explanatory variables: plant shoot mass and percentage root length colonized, total leaf P concentration, total leaf N concentration, and N:P ratio.

Linear regression was used to test for associations between plant and soil factors and emission of VOCs. We tested the amounts of individual EAG-active VOCs as response variables with the following explanatory variables: percentage of root length colonized, plant shoot mass, plant root mass, total leaf P concentration, total leaf N concentration, N:P ratio, and extractable P in the soil.

If AM fungi and P treatments induce changes in the emission of plant VOCs, we need to know whether any aspect of these changes relates to the attractiveness to aphids. Therefore, the association between plant VOCs and the attractiveness to aphids was tested using multiple regressions. The insect host location usually depends on specific combinations and ratios of ubiquitous plant volatiles rather than simple amounts of just one compound (Bruce *et al.*, 2005; Bruce and Pickett, 2011). Therefore we conducted separate analyses using not only the amounts but also the proportions of each compound as explanatory variables and the attractiveness as the response variable. We started with a model that included all possible predictors for plant attractiveness and in subsequent steps we removed those predictors that had low sums of squares of variance accounted for (and high *P* values). The final model was validated by plotting residuals versus fitted values, square root residuals versus fitted values, normal quantile–quantile plots, and constant leverage.

To improve the homogeneity of variance prior to analyses, the percentage of root length colonized was arcsine square root transformed, the plant biomass, total leaf P, total leaf N, N:P ratio, and soil PO_4_ were square root transformed, the amounts of VOCs were log transformed and the proportions of VOCs were arcsine square root transformed. All statistical analysis was performed using SPSS package 20 (IBM).

## Results

### Effect of treatment on plant nutrition (total leaf N and P concentration and N:P ratio) and biomass

**Fig. 1.**
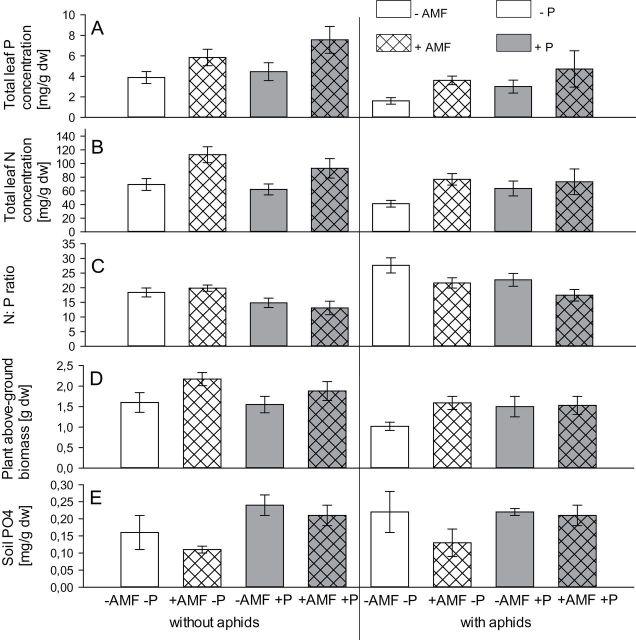
Effect of AM fungi, P addition, and aphids on total leaf P concentration (mg g^–1^ of DW; A); total leaf N concentration (mg g^–1^ of DW; B); leaf N: P ratio (C); plant above-ground biomass (g DW; D), and concentration of soil PO_4_ (mg g^–1^ of DW; E).

Plants had significantly more P in their leaves for both AM fungi and P treatments, but there were no significant treatment interactions (Fig. 1; Table 2). The treatment effect was strongest in response to AM fungi, which resulted in a 40% increase in P concentration (*F*_1,47_=16.6, *P*<0.001). Overall, AM fungi also significantly increased total leaf N concentration by 35% compared with non-mycorrhizal plants (*F*_1,47_=15.27, *P*<0.001). Consequently there was a negative association between AM fungi and N:P ratio, which was 14% lower in mycorrhizal plants (*F*_1,47_=4.29, *P*=0.044). The addition of P to the soil increased leaf P concentrations by 24% (*F*_1,47_=4.21, *P*=0.046) and, as a result, the N:P ratio was 23% higher in the –P compared with the +P treatment (*F*_1,47_=14.20, *P*<0.001). Nutrient concentrations in the leaves were strongly negatively affected by aphids: aphid-infested plants had 42% less total leaf P concentration (*F*_1,47_=18.57, *P*<0.001) and 25% less total leaf N (*F*_1,47_=7.55, *P*=0.009). The N:P ratio in aphid-infested plants was 27% higher in aphid-free plants (*F*_1,47_=19.13, *P*<0.001).

**Table 2.** Results of GLMs for the main effects of AM fungi (–/+), P addition (–/+), aphids (–/+) (n=6), and their two-way and three-way interactions on plant nutrition, plant growth, concentration of soil PO_4_, attractiveness of plant headspace samples to aphids, percentage of root length colonized by AM fungi, and final aphid biomass*P* values <0.05 are highlighted in bold. Direction of effect is indicated by shifts (↑, positive effect; ↓, negative effect). NA, not applicable.

	AM fungi			P			Aphids			AM fungi×P		P×aphids		AM fungi×aphids		AM fungi×P×aphids	
	F		*P*	F		*P*	F		*P*	F	*P*	F	*P*	F	*P*	F	*P*
Total leaf P concentration	↑	16.5	**0.000**	↑	4.2	**0.046**	↓	18.5	**0.000**	0.0	0.894	0.1	0.741	0.0	0.926	0.7	0.401
Total leaf N concentration	↑	15.2	**0.000**		0.05	0.828	↓	7.5	**0.009**	1.9	0.171	2.2	0.143	0.4	0.514	0.5	0.488
N: P ratio	↓	4.2	**0.044**	↓	14.2	**0.000**	↑	19.1	**0.000**	0.5	0.477	0.4	0.557	3.3	0.077	0.6	0.461
Plant shoot mass	↑	7.9	**0.007**		0.07	0.797	↓	7.7	**0.008**	2.1	0.155	1.8	0.186	0.1	0.757	0.4	0.551
Plant root mass		1.7	0.194		2.5	0.124		1.1	0.295	0.3	0.615	0.0	0.879	0.3	0.603	0.9	0.362
Soil PO_4_ concentration		3.6	0.063	↑	10.5	**0.002**		0.4	0.512	0.9	0.357	0.8	0.369	0.1	0.782	0.5	0.506
Attractiveness of plants to aphids	↑	4.7	**0.042**		0.0	0.979		NA	NA	1.2	0.295	NA	NA	NA	NA	NA	NA
AM fungal colonization		NA	NA	↓	7.5	**0.013**		3.9	0.063	NA	NA	4.6	**0.045**	NA	NA	NA	NA
Aphid biomass		0.1	0.732		1.9	0.187		NA	NA	0.8	0.382	NA	NA	NA	NA	NA	NA

There was an overall positive effect of AM fungi on above-ground plant biomass (*F*_1,47_=7.95, *P*=0.007). Plant biomass was not affected by P treatment and was negatively affected by aphids (*F*_1,47_=7.80, *P*=0.008). There were no effects of any of the two-way interactions between AM fungi, aphids, and P on above-ground plant biomass.

### Association between treatment and PO_4_ (plant-available P) in the soil at the end of the experiment

The concentration of PO_4_ in the soil was significantly greater in the +P treatment (*F*_1,47_=10.51, *P*=0.002), where it was 0.22mg g^–1^, than in the –P treatment, where it was 0.15mg g^–1^; approximately 30% less (Fig. 1).

### Effects of treatment on production of plant VOCs

**Fig. 2.**
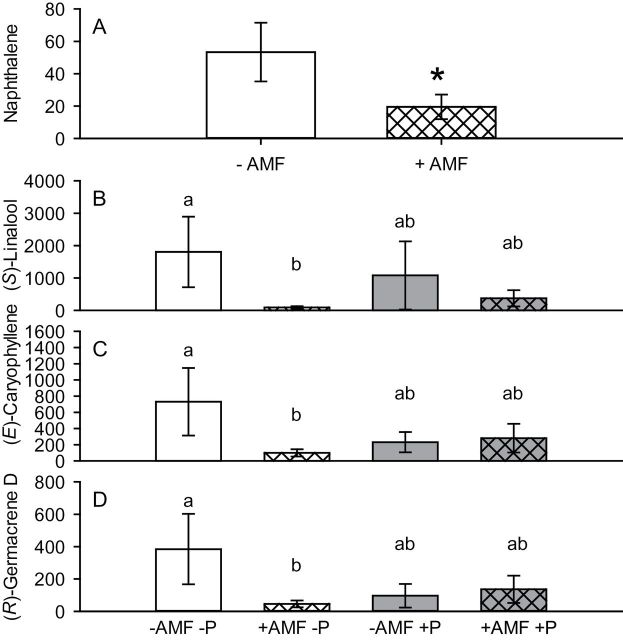
Effect of treatment on emission of plant volatiles (ng g^–1^ of DW 24h^–1^). (A) Overall main effect of AM fungi on emission of naphthalene (there was no significant interaction between AMF and P treatments). (B–D) Effect of interaction between AM fungi and P addition on emission of (*S*)-linalool (B), (*E*)-caryophyllene (C), and (*R*)-germacrene D (D). Bars sharing a letter are not significantly different from each other (*P*>0.05). An asterisk indicates bars that are significantly different (*P*<0.05).

Emissions of all EAG-active VOCs per treatment are shown in Supplementary Table S1 (available at *JXB* online). Significant effects of AM fungi and P treatments on production of plant VOCs are shown in Fig. 2. There was an overall negative effect of AM fungi on emission of naphthalene (*F*_1,17_=6.2, *P*=0.023) (Fig. 2). There was a significant interaction between AM fungi and P treatment associated with the production of (*S*)-linalool (*F*_4,17_=5.65, *P*=0.030), (*E*)-caryophyllene (*F*_4,17_=5.19, *P*=0.037), and (*R*)-germacrene D (*F*_4,17_=4.93, *P*=0.040) (Fig. 2, Supplementary Table S1, available at *JXB* online), where plants with AM fungi but no P produced significantly smaller amounts of these VOCs than the controls. The interaction between AM fungi and P treatment was not significant but was marginal for the sum of all EAG-active VOCs (*F*_4,17_=4.16, *P*=0.058).

### Effect of treatment on attractiveness of plants to aphids

Overall, the attractiveness of mycorrhizal plants to aphids was more than double that of non-mycorrhizal plants (*F*_1,23_=4.71, *P*=0.042): while aphids spent 0.40±0.18min in the bioassay area treated with headspace samples from non-mycorrhizal plants compared with solvent-only bioassay areas, they spent 1.02±0.21min (more than double the time) in the treated area if the arm was treated with headspace samples from mycorrhizal plants compared with solvent-only bioassay areas. There was no association between attractiveness of plants to aphids and P treatment or the interaction between AM fungi and P treatments.

**Fig. 3.**
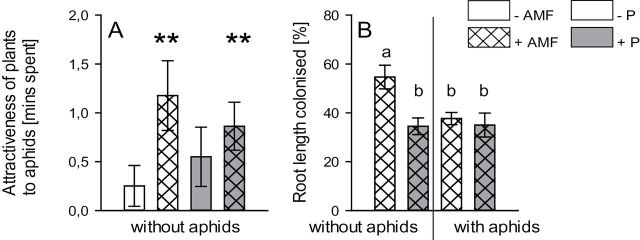
(A) Effect of AM fungi and P additions on attractiveness of plants to aphids. The attractiveness was calculated as time spent by aphids in olfactometer chambers treated with plant headspace samples subtracted by the mean time spent in areas treated with solvent blanks. Asterisks indicate that headspace samples were significantly attractive relative to the solvent blanks. (B) Effect of P additions and aphids on percentage of root length colonized by AM fungi. Bars sharing a letter are not significantly different from each other (*P*>0.05).

Testing whether the headspace samples were attractive or not for each treatment separately showed that mycorrhizal plants were significantly attractive to aphids for –P (*T*=3.32; df=29; *P*=0.002) as well as +P plants (*T*=3.03; df=29; *P*=0.005) (Fig. 3). In contrast, non-mycorrhizal plants did not elicit attraction regardless of P treatment (–P: *P*=0.46; +P: *P*=0.17) (Fig. 3).

### Effect of treatment on percentage of root length colonized by AM fungi

The percentage of root length colonized in plants inoculated with AM fungi plants averaged 40% (range 35–55%), while non-inoculated plants had<1% of root length colonized.

There was an overall negative effect of P on the percentage of root length colonized (*F*_1,23_=7.459, *P*<0.013); –P plants had 46.2±3.6% (mean±standard error) of their root length colonized, while +P plants were 34.7±2.8% colonized. Colonization was also affected by the interaction between P treatment and presence of aphids (*F*_1,23_=4.59, *P*<0.045) (Fig. 3). Plants treated with +aphids and –P had less AM fungal colonization than –aphids –P-treated plants (*P*=0.007). With +P treatment, however, there was no difference in AM fungal colonization between +aphid- and –aphid-treated plants (*P*=0.943). AM fungal colonization of –aphids –P-treated plants was significantly greater than AM fungal colonization in both +aphids +P (*P*=0.003) and –aphids +P (*P*=0.002) treatments.

### Association between the attractiveness of plants to aphids and plant characteristics

**Fig. 4.**
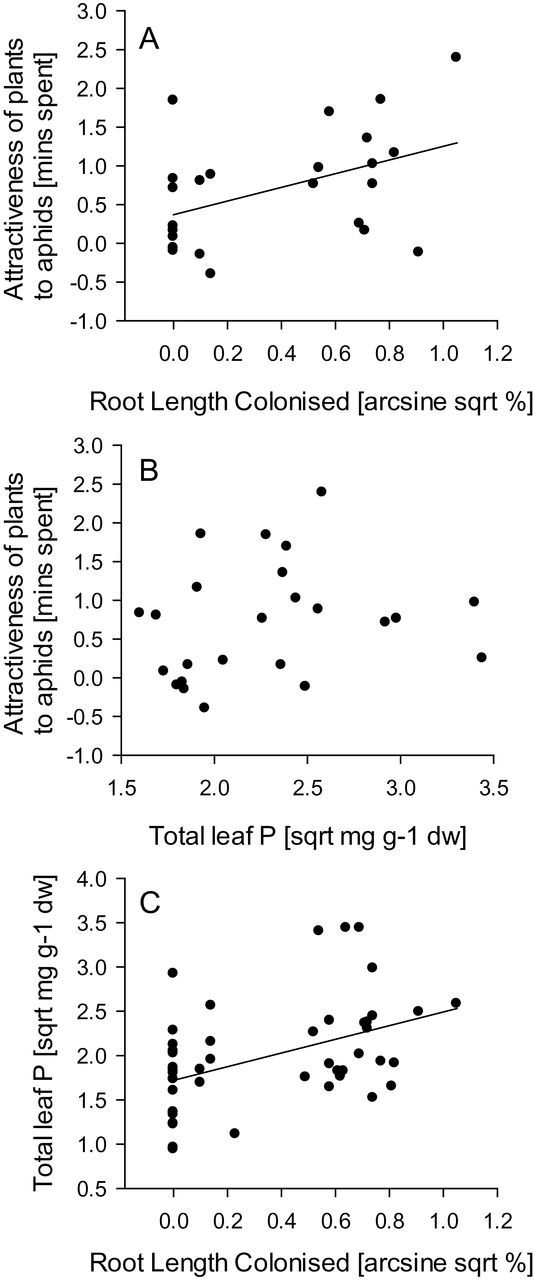
(A) Association between percentage root length colonized by AM fungi and attractiveness of plants to aphids (*R* ^2^=0.19; *F*_1,23_=5.37, *P*=0.030). (B) Association between total leaf P concentration and attractiveness of plants to aphids (*R* ^2^=0.04; *F*_1,23_=0.99, *P*=0.330). (C) Association between percentage root length colonized by AM fungi and total leaf P concentration (*R* ^2^=0.20, *F*_1,46_=11.48, *P*=0.001).

The attractiveness of plants to aphids was positively associated with percentage root length colonized by AM fungi (*R* ^2^=0.19; *F*_1,23_=5.37, *P*=0.030; Fig 4). There was no association between the attractiveness of plants to aphids and total leaf P (*P*=0.330; Fig 4), total leaf N (*P*=0.142), N:P ratio (*P*=0.672), concentration of PO_4_ in the soil (*P*=0.585), plant shoot mass (*P*=0.308) or root mass (*P*=0.120).

The percentage of root length colonized by AM fungi was negatively associated with the concentration of PO_4_ in the soil (*R* ^2^=0.09, *F*_1,46_=4.57, *P*=0.038) and positively associated with total leaf P concentration (*R* ^2^=0.20, *F*_1,46_=11.48, *P*=0.001) (Fig. 4). In addition, there was a positive association between total leaf P concentration and total leaf N concentration (*R* ^2^=0.72, *F*_1,46_=114.9, *P*<0.001), suggesting that our experimental plants were not P or N limited. Plant shoot mass was positively associated with the percentage of root length colonized (*R* ^2^=0.21, *F*_*1*,46_=12.33, *P*=0.001) (Fig. 4), total leaf P concentration (*R* ^2^=0.16, *F*_1,46_=8.46, *P*=0.006) and total leaf N concentration (*R* ^2^=0.25, *F*_1,46_=15.12, *P*<0.001).

### Association between plant characteristics and emission of VOCs

The amounts of EAG-active VOCs emitted used in the analysis were calculated per unit of dry mass (g^–1^ of DW) so that the effect of plant shoot mass on production of plant VOCs was not related to plant size directly.

Plant shoot mass was negatively associated with emission of (*E*,*E*)-4,8,12-trimethyl-1,3,7,11-tridecatetraene (*R* ^2^=0.29, *F*_1,46_=8.81, *P*=0.007) and (*E*)-caryophyllene (*R* ^2^=0.29, *F*_1,46_=8.96, *P*=0.007). A further negative association between plant shoot mass and emission of (*R*)-germacrene D was non-significan, but marginal (*R* ^2^=0.16, *F*_1,46_=4.10, *P*=0.055).

Total leaf P was negatively associated with emission of naphthalene (*R* ^2^=0.21, *F*_1,46_=6.04, *P*=0.022). Total leaf N was also negatively associated with emission of naphthalene (*R* ^2^=0.18, *F*_1,46_=6.78, *P*=0.042).

### Associations between the attractiveness of plants to aphids and plant VOCs

There was no significant association between the attractiveness of plants to aphids and the amounts of individual VOCs, but there was a highly significant (*R* ^2^=0.66, *F*_6,23_=5.336, *P*=0.003) association between attractiveness and proportions of: (*Z*)-2-hexenal (*T*=3.42, *P*=0.003), (*Z*)-2-heptenal (*T*=–2.30, *P*=0.034), 6-methyl-5-hepten-2-one (*T*=4.15, *P*=0.001), (*R*, *S*)-β-pinene (T=–2.45, *P*=0.025), (*E*)-caryophyllene (T=4.31, *P*<0.001), and (*E*)-β-farnesene (*T*=4.06, *P*<0.001). There was a marked reduction in total VOC emission for +AM and +P treatments (Supplementary Table S1, available at *JXB* online).

## Discussion

The key aim of this study was to improve our understanding of how AM fungi regulate the attractiveness of plants to aphids. A previous study (Babikova *et al.*, 2013a) found that bean plants colonized by AM fungi were more attractive to pea aphids than were bean plants without AM fungi. Here, we tested the hypothesis that attractiveness is a direct response of increased P uptake by mycorrhizal plants. Although we observed positive effects of AM fungi on the attractiveness of plants to aphids, we found no evidence that attractiveness was regulated by P supply. This result implies that P supply *per se* is not the main driver through which AM fungi influence the attractiveness of plants to aphids.

We found that AM fungi had positive effects on total leaf P and total leaf N concentrations and that P addition positively affected total leaf P concentrations, suggesting that the treatments were effective in influencing plant nutrition. Under the hypothesis that enhanced leaf P is the key mechanism by which AM fungi increase the plant attractiveness to aphids, we predicted that plants with more P should be more attractive to aphids and, specifically, that non-mycorrhizal plants supplied with extra P would have similarly enhanced attractiveness to aphids as mycorrhizal plants with no added P. However, there was no evidence that +P-treated plants were more attractive to aphids than –P-treated plants. It is possible that this result might be due to the slightly weaker effect of the +P treatment on leaf P concentrations in aphid-free non-mycorrhizal plants compared with the effect of inoculation with AM fungi. However, this is unlikely since, irrespective of treatment group, we did not find any correlation between the attractiveness of plants to aphids and leaf P concentrations (or N concentrations). In contrast, mycorrhizal plants were more attractive to aphids and, irrespective of treatment, the percentage of root length colonized by AM fungi was positively associated with plant attractiveness to aphids. In our experiment, AM fungi are therefore key drivers of aphid host location in this interaction, and the mechanism of the increased attractiveness of mycorrhizal plants to aphids (Babikova *et al.*, 2013a) involves other aspects of colonization of plants by AM fungi than simply improved plant P acquisition.

We have shown that AM fungi substantially increase plant P acquisition and attractiveness of plants to aphids, but that the increased attractiveness of plants to aphids is unlikely to be a direct consequence of increased P uptake. Instead, induced changes in emission of plant VOCs, which make plants more attractive to aphids, could be regulated by aspects of mycorrhizal colonization other than P uptake. The mechanism is likely to operate via AM fungi-induced defence-related systemic signalling, which leads to altered production of plant VOCs that have role in aphid host location.

Aphid attractiveness is largely determined by their ability to use chemical cues released by plants. In order to test the effect of our treatments on production of plant VOCs, we quantified the compounds that have been shown previously to elicit EAG activity with antennae of pea aphids (Babikova *et al.*, 2013b). We found that the emission of the aromatic hydrocarbon naphthalene was significantly suppressed as a response to AM fungi. Furthermore, the emission of (*S*)-linalool, (*E*)-caryophyllene, and (*R*)-germacrene D were regulated by an interaction between AM fungi and P addition. Emission of all these compounds was significantly suppressed in response to AM fungi; however, this effect was seen only in the –P treatment. In the +P treatment, the quantities emitted were not affected by AM fungi treatment and this could be because mycorrhizal colonization is usually affected negatively by high P availability (Gange *et al.*, 1999). It is therefore possible that the combined effect of AM fungi and P addition on emission of these VOCs was not significant because the plants were colonized by AM fungi significantly less compared with plants where no extra P was supplied.

The effect of the treatments on VOCs emissions is consistent with the carbon–nutrient balance hypothesis (Chapin, 1980), which suggests that nutrient deficiency limits plant growth more than it limits the rate of photosynthesis. Under nutrient limitation, plant growth rate is reduced and photosynthesis remains unchanged, leading to accumulation of carbohydrates, which may then become substrates for the fabrication of secondary metabolites, e.g. terpenoids (Gershenzon, 1994). Although the abundance of the substrate is only one of many factors limiting biosynthesis of secondary metabolites (Gershenzon, 1994), it may potentially lead to enhanced emission of terpenoids in plants with limited nutrients, such as those without mycorrhiza and/or with no added P.

We showed that plants alter their production of VOCs [naphthalene, (*S*)-linalool, (*E*)-caryophyllene, and (*R*)-germacrene D] in response to AM fungi and P treatments. We found that the attractiveness of plants to aphids was associated with the proportions of six VOCs: (*Z*)-2-hexenal, (*Z*)-2-heptenal, 6-methyl-5-hepten-2-one, (*R*, *S*)-β-pinene, (*E*)-caryophyllene, and (*E*)-β-farnesene. Furthermore, total volatile emission was reduced in mycorrhizal plants. Hence, the mechanism of pea aphid host location is likely to rely on specific ratios of these volatiles, as suggested by previous findings that used different plant–insect systems (Birkett *et al.*, 2004; Bruce and Pickett, 2011). Mycorrhizal plants were more attractive to aphids and also emitted less (*E*)-caryophyllene than non-mycorrhizal plants, while the attractiveness of plants to aphids was associated with higher proportions (note that this is not necessarily higher amounts) of (*E*)-caryophyllene. However, this association with attractiveness was significant only in the multiple regression analysis, which included all six compounds together. The simple regression of (*E*)-caryophyllene alone did not reveal a significant association with attractiveness of plants to aphids (data not shown). The proportions of these six compounds individually (within the blend of all EAG-active VOCs) were not significantly affected by treatment (data not shown), suggesting that small, statistically insignificant, changes of amounts or proportions of these compounds in relation to each other is what affects aphid behaviour (see also Birkett *et al.*, 2004; Pareja *et al.*, 2009; Bruce and Pickett, 2011).

Changes in emissions of any compound (in response to treatment) will necessarily affect the proportions of all other compounds emitted. These changes could therefore provide an alternative mechanism for pea aphid host location; however, there are a large number of combinations of proportions to test for. In addition, some compounds could be substituted by others of similar molecular structure by insects to elicit the same response (Bruce and Pickett, 2011), which may further complicate the relationship between VOCs and insect host location. Nevertheless, unravelling which key compounds drive insect behavioural response in the field should be better determined using wild populations of plants on which the insect initially evolved, as planted cultivars may have altered chemistry (Gols *et al.*, 2011; Tamiru *et al.*, 2011) and may hence also emit different volatile compounds.

As predicted, plants had less mycorrhizal colonization of their roots when supplied with P and we also found that aphids had a negative effect on mycorrhizal colonization. The antagonistic effect of aphids could operate either via reduced carbon allocation to AM fungi, because aphids drain carbon from the plants, or by defence-related signalling induced by the aphids that is antagonistic to AM fungi. We predicted that the antagonistic effects of increasing P availability and of aphids on mycorrhizal fungal colonization would be additive, but this prediction was not supported by our data. Instead, the antagonistic effect of aphids on AM fungal colonization was the same with or without added P, suggesting that plants with access to greater soil P can compensate for the negative impacts of aphids on AM fungi.

Aphid fecundity is often significantly less on plants with lower nutritional quality (Grüber and Dixon, 1988). Because herbivorous insects generally perform better on plants with greater concentrations of N and P in leaves (Elser *et al.*, 2000; Denno and Fagan, 2003; Woods *et al.*, 2004; Vannette and Hunter, 2009), and mycorrhizal plants often contain more P in their tissues (Vannette and Hunter, 2009; Ryan *et al.*, 2012; Walder *et al.*, 2012), increased P concentration in mycorrhizal plants should be predicted to have positive effects on insect growth (Schoonhoven *et al.*, 2005; Vannette and Hunter, 2009). Despite this prediction, we found no effect of treatment on aphid final biomass. It is possible, however, that the effect of treatment on aphid fecundity was counterbalanced by changes in the weight of individual aphids (e.g. it remains possible that more, but smaller, aphids were produced) but we do not have the data on aphid numbers to test this. Thus, improved nutrition did not lead to increased aphid total biomass in our study; hence plants that are more attractive may not necessarily improve aphid growth.

Although our results contribute to our understanding of how land-management practices may affect interactions among AM fungi, plants, and aphids, additional experiments are required to test how the myriad additional edaphic and environmental factors found in nature (e.g. Gouinguené and Turlings, 2002) affect these interactions. Moreover, we need to understand better the spatial scales over which VOCs are utilized under field conditions by organisms from different trophic levels (Gols *et al.*, 2011). While this needs to be determined in a field study, our findings might suggest that fertilizer additions of P will have no measurable effect on attractiveness of plants to aphids in the field. At the same time, some agricultural practices affect AM fungal development (Lekberg and Koide, 2005), which might impact on attractiveness of plants to aphids. In addition, promotion of AM fungal development might lead to formation of common mycelial networks that connect individual plants. These networks can be conduits for transfer of both nutrients (Walder *et al.*, 2012) and warning signals of aphid attack between the plants that affect the attractiveness of plant to aphids and their natural enemies (Babikova *et al*., 2013*b*, *c*). Therefore, experiments investigating how mycorrhizal fungi regulate aphid populations in agro-ecosystems need to carefully consider all aspects of mycorrhizal functioning.

## Supplementary data

Supplementary data are available at *JXB* online.

Supplementary Table S1. Production of volatile organic compounds that elicit electrophysiological activity on antennae of pea aphids (*Acyrthosiphon pisum* Harris), by broad beans (*Vicia faba* L.) in response to phosphorus and arbuscular mycorrhizal fungal treatment.

Supplementary Data

## Abbreviations:

AM: arbuscular mycorrhiza
DW: dry weight
EAG: electroantennography
GC: gas chromatography
GLM: general linear models
N: nitogen
P: phosphorus
VOC: volatile organic compound.
