# Supplementary material for: Increasing phosphorus supply is not the mechanism by which arbuscular mycorrhiza increase attractiveness of bean (Vicia faba) to aphids
Source: J Exp Bot. 2014 Jul 8;65(18):5231–41. doi: 10.1093/jxb/eru283 (PMC4157707; doi:10.1093/jxb/eru283)
Supplement: Supplementary Data [file supp_65_18_5231__index.html]

Increasing phosphorus supply is not the mechanism by which arbuscular mycorrhiza increase attractiveness of bean (Vicia faba) to aphids — Increasing phosphorus supply is not the mechanism by which arbuscular mycorrhiza increase attractiveness of bean (Vicia faba) to aphids — Supplementary Data 

# Increasing phosphorus supply is not the mechanism by which arbuscular mycorrhiza increase attractiveness of bean (*Vicia faba*) to aphids

## Supplementary Data

Data files

**Files in this Data Supplement:**

- Supplementary Data - Supplementary Data
