# Supplementary material for: Increasing phosphorus supply is not the mechanism by which arbuscular mycorrhiza increase attractiveness of bean (Vicia faba) to aphids
Source: J Exp Bot. 2014 Jul 8;65(18):5231–41. doi: 10.1093/jxb/eru283 (PMC4157707; doi:10.1093/jxb/eru283)
Supplement: Supplementary Data [file supp_eru283_jexbot121772_file001.pdf]

Table S1. Production of volatile organic compounds [ng g^{-1} dw $24h^{-1}$], which elicit electrophysiological activity on antennae of pea aphids (*Acyrthosiphon pisum* Harris), by broad beans (*Vicia faba* L.) in response to phosphorus (+/– P) and arbuscular mycorrhizal fungal (+/– AM fungi) treatment. Kovats index is the retention time of elution of the compound normalised to the retention times of a series of *n*-alkanes. Asterisks indicate significant effects of treatment on emission ($P < 0.05$; see Fig. 2 and text for details).

Volatile compounds	Kovats index	Treatment			
		–P –AM fungi	–P +AM fungi	+P –AM fungi	+P +AM fungi
(*Z*)-2-Hexenal	817	4.08 ± 2.92	3.52 ± 1.87	3.11 ± 1.46	1.98 ± 0.67
(*E*)-2-Hexenal	825	1.72 ± 1.72	0.83 ± 0.83	0.52 ± 0.52	0.91 ± 0.45
(*E,E*)-2,4-Hexadienal	880	39.4 ± 21.6	25.5 ± 15.9	52.8 ± 48.4	16.2 ± 12.8
(*Z*)-2-Heptenal	924	129 ± 79	104. ± 50.9	189 ± 173	62.9± 48.5
Benzaldehyde	929	500 ± 253	97.3 ± 33.3	269 ± 240	124 ± 60.5
6-Methyl-5-hepten-2-one	967	16.2 ± 5.91	10.1 ± 7.0	16.5 ± 15.0	6.10 ± 3.28
(*R, S*)-*β*-Pinene	972	39.4 ± 20.2	6.42 ± 2.51	22.2 ± 20.1	7.65 ± 4.48
(*Z*)-3-Hexenyl acetate	986	77.4 ± 32.1	40.9 ± 9.2	45.4 ± 19.9	45.0 ± 22.1
(*S*)-Linalool *	1086	1806 ± 1088	93.1 ± 36.9	1080 ± 1052	374 ± 251
Naphthalene *	1168	57.9 ± 24.9	29.3 ± 13.9	48.9 ± 28.5	9.9 ± 4.8
Methyl salicylate	1172	16.2 ± 13.6	6.22 ± 4.38	4.42 ± 3.32	1.90 ± 1.65
Cinnamaldehyde	1232	146 ± 64.4	50.1 ± 27.8	154 ± 137	12.4 ± 6.5
(*E*)-*C*aryophyllene *	1424	731 ± 417	99.4 ± 44.7	231 ± 125	281 ± 177
(*E*)-*β*-Farnesene	1450	7.22 ± 3.21	2.58 ± 0.88	9.85 ± 8.71	1.52 ± 0.79
(*R*)-Germacrene D *	1486	385 ± 218	45.8 ± 21.1	96.3 ± 73.0	136 ± 84.5
(*E,E*)-4,8,12-Trimethyl-1,3,7,11- tridecatetraene	1570	109 ± 34.2	55.1 ± 8.8	79.1 ± 35.8	93.4 ± 50.7
Total production		4051 ± 2033	665 ± 185	2298 ± 1900	1174 ± 630
